# Synergistic Effect of Anti-Angiogenic and Radiation Therapy: Quantitative Evaluation with Dynamic Contrast Enhanced MR Imaging

**DOI:** 10.1371/journal.pone.0148784

**Published:** 2016-02-10

**Authors:** Hyun Jung Koo, Myoungsun Lee, Jin Kim, Chul Woong Woo, Seong-Yun Jeong, Eun Kyung Choi, Namkug Kim, Jin Seong Lee

**Affiliations:** 1 Research Institute of Radiology and Department of Radiology, University of Ulsan College of Medicine, Asan Medical Center, Seoul, Republic of Korea; 2 Asan Institute for Life Sciences, Asan Medical Center, Seoul, Republic of Korea; 3 Department of Radiation Oncology, University of Ulsan College of Medicine, Asan Medical Center, Seoul, Republic of Korea; Technische Universitaet Muenchen, GERMANY

## Abstract

**Purpose:**

We assessed the effects of anti-angiogenic therapy (AAT) on radiation therapy (RT), evaluating the tumor growth and perfusion patterns on dynamic contrast enhanced MR (DCE-MR) images.

**Methods:**

Thirteen nude mice with heterotopic xenograft cancer of human lung cancer cell line were used. To observe the interval change of the tumor size and demonstrate the time-signal intensity enhancement curve of the tumor, the mice were subdivided into four groups: control (n = 2), AAT (n = 2), RT (n = 5), and combined therapy (AART, n = 4). DCE-MR images were taken four weeks after treatment. Perfusion parameters were obtained based on the Brix model. To compare the interval size changes in the RT group with those in the AART group, repeated measures ANOVA was used. Perfusion parameters in both the RT and AART groups were compared using a Mann-Whitney U test.

**Results:**

Tumor growth was more suppressed in AART group than in the other groups. Control group showed the rapid wash-in and wash-out pattern on DCE-MR images. In contrast to RT group with delayed and prolonged enhancement, both AAT and AART groups showed the rapid wash-in and plateau pattern. The signal intensity in the plateau/time to peak enhancement (P<0.016) and the maximum enhancement ratio (P<0.016) of AART group were higher than those of RT group.

**Conclusions:**

AART showed synergistic effects in anticancer treatment. The pattern of the time-intensity curve on the DCE-MR images in each group implies that AAT might help maintain the perfusion in the cancer of AART group.

## Introduction

The main goal of radiation therapy (RT) is local cancer control for a better outcome. To improve the cure rates or reduce side effects, the combination treatment of RT and pharmaceutical agents such as anti-angiogenic (AA) agents or anti-vascular drugs is being used and clinically investigated [1, 2]. However, the mechanism of the enhanced effect of the combination therapy of RT and drugs such as AA agents is still unclear.

AA agents can induce primary tumor shrinkage by pruning the formation of immature tumor vessels. Because RT can cause cell injury by inducing oxygen-free radicals in tumor cells, oxygen is essential for achieving the anti-cancer effect of RT. However, RT can induce extensive necrosis especially in the central portion of a tumor. Necrosis with decreased blood supply causes a hypoxic condition of the tumor and eventually decreases the tumoricidal effect of RT by reducing the oxygen-free radicals [3–6].

The hypothesis that AA therapy (AAT) may help normalize the vascular structures of a tumor and promote improved blood perfusion was recently suggested [7]. The hypoxic area in the tumor may eventually decrease, and the effect of RT could be optimized. The perfusion changes can be demonstrated on dynamic contrast enhanced-magnetic resonance (DCE-MR) images by obtaining the time-signal intensity curves and analyzing the perfusion parameters [1, 8, 9]. For example, high values of the peak enhancement and the time to peak enhancement (TTP) of a tumor have been shown after RT, compared to those of non-irradiated tumors [10]. This finding suggests that the prolonged enhancement and delayed wash-out pattern of the contrast materials were due to the increased extracellular extravascular space, i.e., the necrotic component, and the decreased vascular permeability of the irradiated tumor [10].

In this study, we demonstrated and evaluated the tumor growth and perfusion changes induced by RT and the combination therapy of RT and an AA agent in a murine cancer model. With DCE-MR imaging, we might help to draw insights into the synergistic effect of the combination therapy of AAT and RT.

## Methods

The Institutional Animal Care and Use Committee in Asan Medical Center approved this study, and all the experiments were performed in accordance with the committee guidelines. Animal care and treatment were guided according to the 8^th^ edition of the Guide for the Care and Use of Laboratory Animals.

### Animal and experimental protocol

Thirteen nude mice were implanted with A549 heterotopic xenograft cancer cells from the human lung cancer cell line (CCL-185TM; American Type of Culture Collection, Manassas, VA, USA), which was cultivated in Dulbecco’s modified Eagle’s Medium (DMEM, GIBCO, Carlsbad, CA, USA) mixed with 10% heat-inactivated FBS and 1% streptomycin-neomycin (GIBCO) at 37°C in a humidified CO_2_ environment. With the harvested amount of 3 x 10^6^ cancer cells, a total volume of 50 μL of Hank’s Balanced Salt Solution (HBSS; GIBCO) was implanted in the left thigh muscles of the seven-week-old male BALB/c nude mice (OrientBio, Sungnam, Korea).

Each mouse was randomly assigned to one of the following four groups: (1) the control group (n = 2); (2) AAT group (n = 2); (3) RT group (n = 5); and (4) the combination therapy (AART) group (n = 4). The second-generation multi-targeted receptor tyrosine kinase inhibitor, SU11248 (sunitinib malate, Pfizer Inc., New York, NY, USA), inhibits cellular signaling by targeting the receptors of the platelet-derived growth factor (PDGF) and the vascular endothelial growth factor (VEGF), which have important roles in cancer angiogenesis and proliferation. Using this drug, an experimental trial for the evaluation of the additional effect of an AA agent on RT was performed. In the AAT group, 25 mg/kg/day of SU11248 with a dimethyl sulfoxide (DMSO, GIBCO, Carlsbad, CA, USA) solution was given orally. For the control group and the RT group, the DMSO solution was given as a placebo. The tumor was subjected to the scheduled RT (8x2.5 Gy, three times/week) three times a week from Day 0 while the other body parts were shielded (Clinac 21EX, Varian Medical Systems, Palo Alto, CA, USA).

The tumor diameters (length and width) were measured with calipers on Days 0, 2, 4, 7, 9, 11, 14, 16, 18, 21, 23, and 25. The tumor growth in each group was evaluated by measuring the volumes (width^2^ x length/2) of the tumors on a given day [1]. The tumor volume on a given day was calculated as the ratio of the initial volume (V_0_) at the onset of the treatment (Day 0) to the volume of the given day. To avoid potential adverse events related to the expected outcome, all tumors should not exceed 20 mm in a longest diameter and be no more than 10% body weight.

Mice were fed with sterilized food and housed in laminar flow filtered hoods. All mice were monitored their conditions twice per day. We have planned to euthanize for any mice found to be moribund, cachectic, lethargic, bleeding from the tumor, or with any conditions unable to eat or drink. However, there were no severely ill animals prior to the endpoint of the study. Two mice died during the anesthesia for MR acquisition. On the 25^th^ day, thirteen mice underwent MR imaging followed by euthanasia using carbon dioxide.

### MR imaging technique

The shortening of the T1-relaxation time by the contrast agent enhanced the tissue. The T1-weighted gradient-echo was also used in the DCE-MR imaging to estimate the T1-relaxation rate and to measure the contrast agent concentration. The baseline DCE MR images at the start of the experiment and the follow-up image were obtained after four weeks in each group. The DCE-MR images of the right thigh tumor masses were obtained using a 4.7-T MR scanner (Biospec; Bruker Medical System, Karlsruhe, Germany) and a 30 mm, single-tuned surface coil. The transverse T1-weighted dynamic MR images were acquired for 12 minutes and 48 seconds (repetition time/echo time = 60.0/4.5 msec; flip angle = 70°; slice thickness = 1 mm; field of view = 30x30 mm; and matrix size = 128x128). After the four initial images were obtained as the baseline images, a contrast agent (gadoteric acid, Gd-DOTA, Guerbet, Roissy CdG, France) was intravenously injected into the tail vein of each mouse. In all the mice, 3 μmol/g (6 μL/g) of gadoteric acid was administered at a flow rate of 129 mL/hr. One hundred continuous dynamic MR images were acquired every 7.6 seconds for 12 minutes and 48 seconds. During the MR imaging, anesthesia was maintained using 1.5% isoflurane in a 1:2 mixture of O_2_/N_2_O. Gadoteric acid was intravenously injected with a 25-gauge needle into the tail vein. A heated pad was placed under each mouse to maintain its body temperature during the MR examination.

### MR image analysis and perfusion parameters

**Fig 1:**
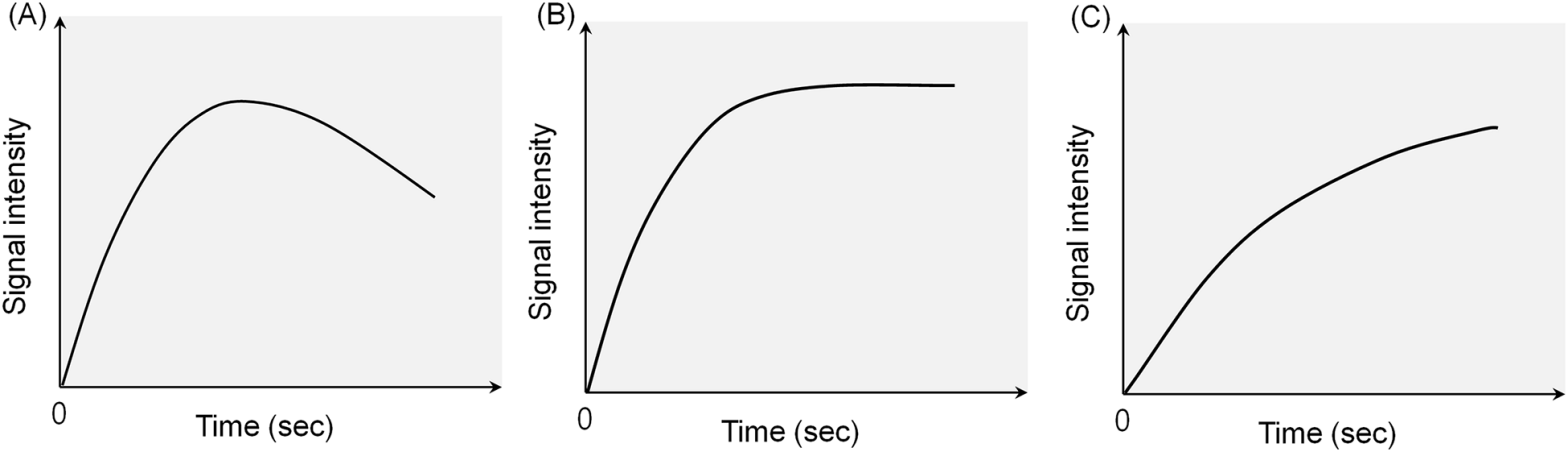
Patterns of the time-intensity curves demonstrating the perfusion state of a tumor on dynamic contrast-enhanced MR images. (A) Pattern I: Rapid wash-in followed by the washout phase. (B) Pattern II: Rapid wash-in followed by a plateau after the peak enhancement. (C) Pattern III: Delayed wash-in and prolonged enhancement.

The tumor time-signal intensity curve of each MR image was drawn with dots that indicated the signal intensities of the 100 continuous MR images of each mouse. Four initial images were marked at the starting point, and the other signal intensities were counted based on the ratio of the initial point to the following points. The time-signal intensity curves were obtained and interpreted according to the wash-in and wash-out patterns (Fig 1); for example, Pattern I, the rapid wash-in followed by wash-out phase; Pattern II, rapid wash-in followed by a plateau after the peak enhancement; and Pattern III, delayed wash-in and prolonged enhancement. Similarly, a previous report that evaluated the perfusion changes in seven patients with unresectable hepatocellular carcinoma using DCE-MR imaging after treatment with the AA agent thalidomide showed a significant change in the enhancement slope of the time-intensity curve after the treatment [11].

In this study, the Brix model [12, 13], a linear two-compartment open model where in the peripheral compartment has negligible effects on the central compartment, was used because it did not need T10 mapping or arterial input function measurements. In the Brix model, with the S_0_ (baseline signal intensity), TA (time of arrival), and A^H^ (the constant that corresponded to the size of the interstitial space), we obtained each parameter according to the following formula.

$$\text{S}\left(\text{t}\right)/{\text{S}}_{0}=\;1+{\text{A}}^{\text{H}}\times {\text{K}}_{\text{ep}}\times [({\text{exp}}^{-\text{Kep}\;(\text{t}-\text{TA})}\;-\;{\text{exp}}^{-\text{Kel}(\text{t}-\text{TA})})/({\text{K}}_{\text{el}}-{\text{K}}_{\text{ep}})]$$

As the concentration of the contrast agents decreased in the plasma due to the renal excretion of the contrast, the contrast agents diffused back into the plasma from the interstitial space in the target lesion. This phenomenon is represented by the rate constant K_ep_ (sec ^-1^, exchange rate constant from the extracellular space back to the plasma in the vascular structure, K_ep_ = K_trans_ / v_e_, in the Tofts model parameters) [14]. K_el_ (sec ^-1^, the elimination rate constant from the plasma to the renal excretion, although some contrast agents show hepatic excretion) is the elimination coefficient used to denote the amount of the renal excretion of contrast from the plasma. This model showed dynamic tissue responses after the intravenous bolus contrast injection. S_plat_ (the signal intensity in the plateau), TTP (time to peak enhancement), and MER (maximum enhancement ratio) were also analyzed by an in-house program developed using Matlab^™^ (The Mathworks, Inc., Natick, MA, USA).

Two radiologists measured the five quantitative perfusion parameters, i.e., K_ep_, K_el_, AH, S_plat_/TTP, and MER, in the regions of interest (ROIs) of the tumors all together, and the ROIs were placed within the tumors to avoid overlap with normal tissue and the far peripheral zone. Because oxygen can be supplied to the peripheral zone of a tumor through diffusion and without adequate vascular supply, necrosis is usually initiated in the central portion of the tumor. According to this concept, the central area was selected for the estimation of the effect of the AA agent, and the ROIs were located in the tumors, 2 mm from their peripheral margin. Each ROI was obtained three times, and the average value of each parameter was calculated.

### Statistical analysis

To show the interval increase in the tumor size, the control, AAT, RT, and AART groups were used. The ratio of the tumor growth in each group on the follow-up dates was plotted as median and interquartile range (25^th^– 75^th^ percentile). To compare the interval size changes in the RT group with those in the AART group, repeated measures ANOVA was used. Due to the small sample size, to evaluate the additive effect of the combination therapy, the parameters obtained based on the Brix model from the T1-weighted DCE-MR images in both the RT and AART groups were analyzed using a Mann-Whitney U test (SPSS 20.0 version, SPSS Inc., Chicago, IL, USA). In the statistical analysis, a value was considered significant when the *p* value was less than 0.05.

## Results

### Tumor growth rate

**Fig 2:**
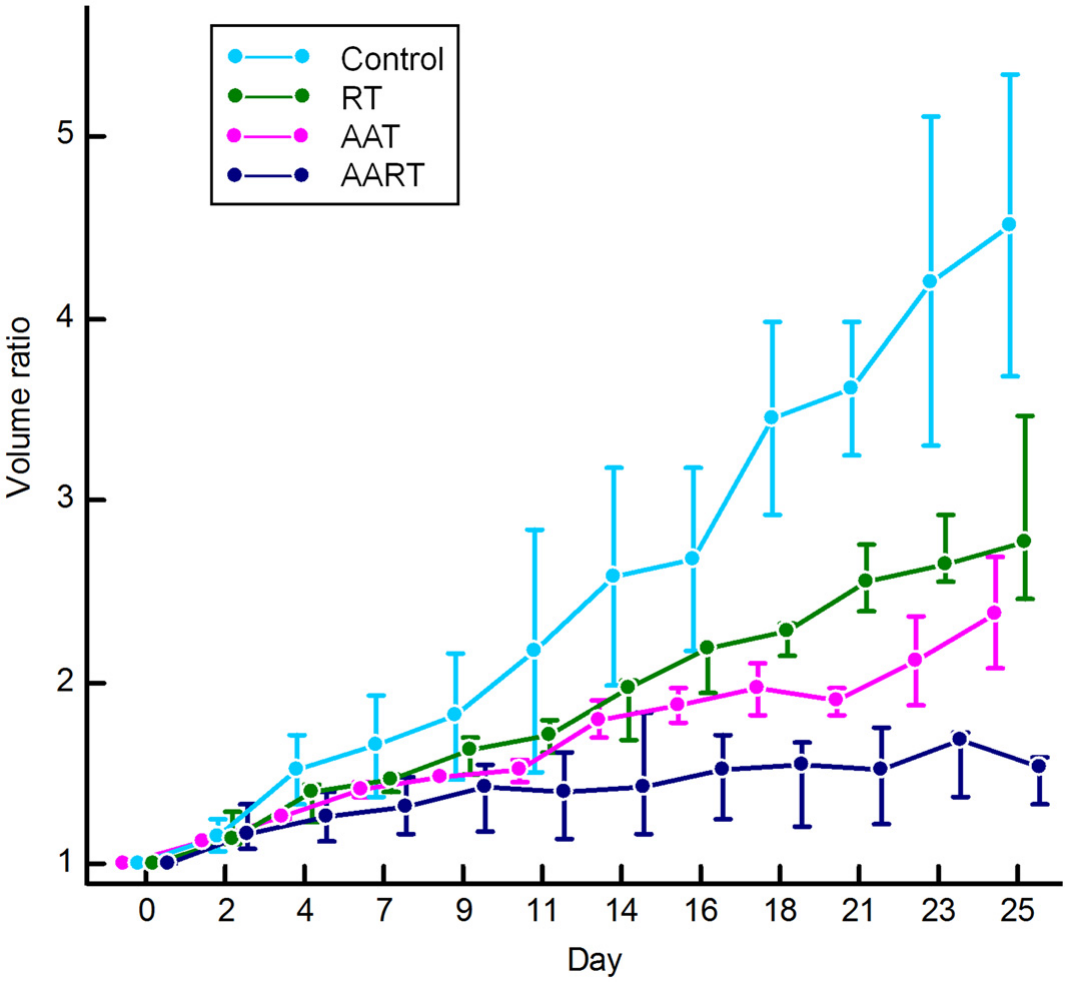
Tumor growth curves obtained by measuring the volumes of the tumors in the study groups. The volume ratio of the tumor was obtained from the initial volume (V_0_) at the onset of the treatment (Day 0) to the following day. On the tumor growth curve, the mean volume ratio in each group on the follow-up dates was plotted. In the AART group, the tumor growth rate is much more suppressed than those in the other groups. RT, radiation therapy; AAT, antiangiogenic agent therapy; and AART, AAT plus RT.

The maximum volume of the tumors was 780 mm^3^ in the control group (mean 215 mm^3^ for all time points), and the maximum diameter of the tumors was 13.5 mm. All measured serial volumes of the tumors are demonstrated in S1 Table. The tumor growth rate was more suppressed in the group treated with AART (n = 4) than in the RT alone group (n = 5) (p<0.001) (Fig 2). Moreover, compared to the control group that showed an increase in the tumor volume, which was measured at the last follow-up day, approximately five times that in the initial state, the AART group showed approximately only a two-fold increase from the initial state.

### MR images and enhancement-time curve

**Fig 3:**
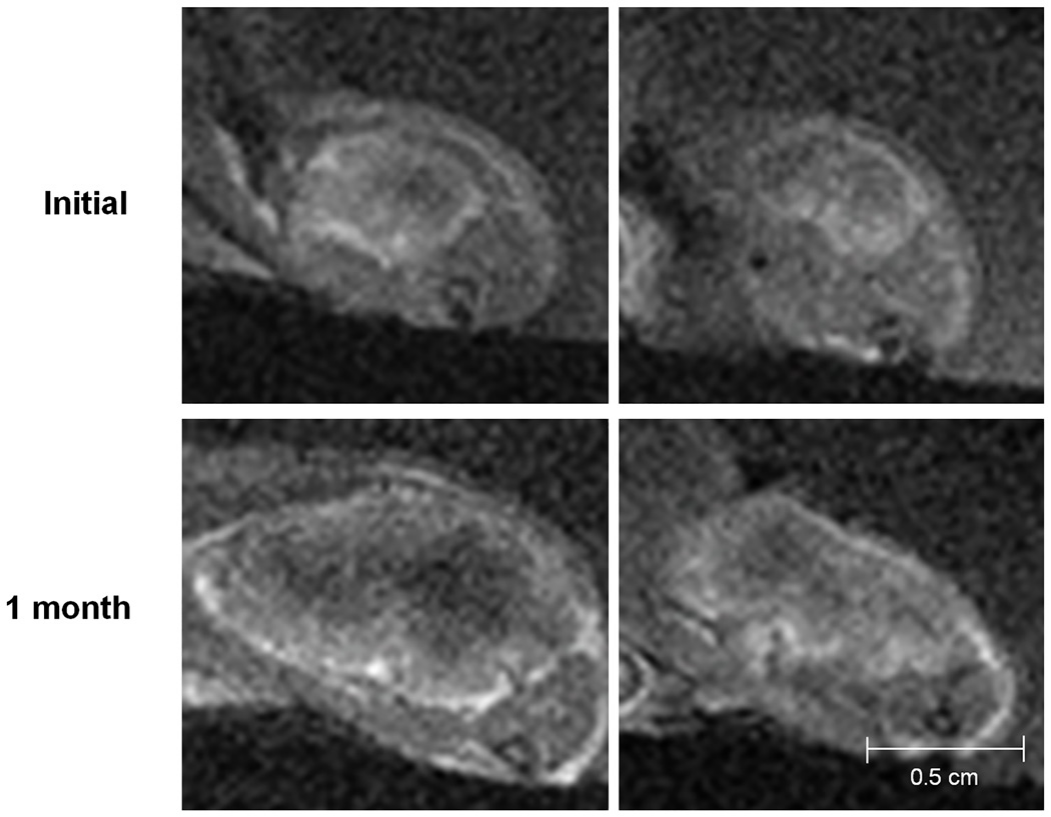
Initial and post-treatment MR images of the control group. The sizes of the tumors increase and show low signal intensity changes in the central portion of the tumors that indicate necrosis.

**Fig 4:**
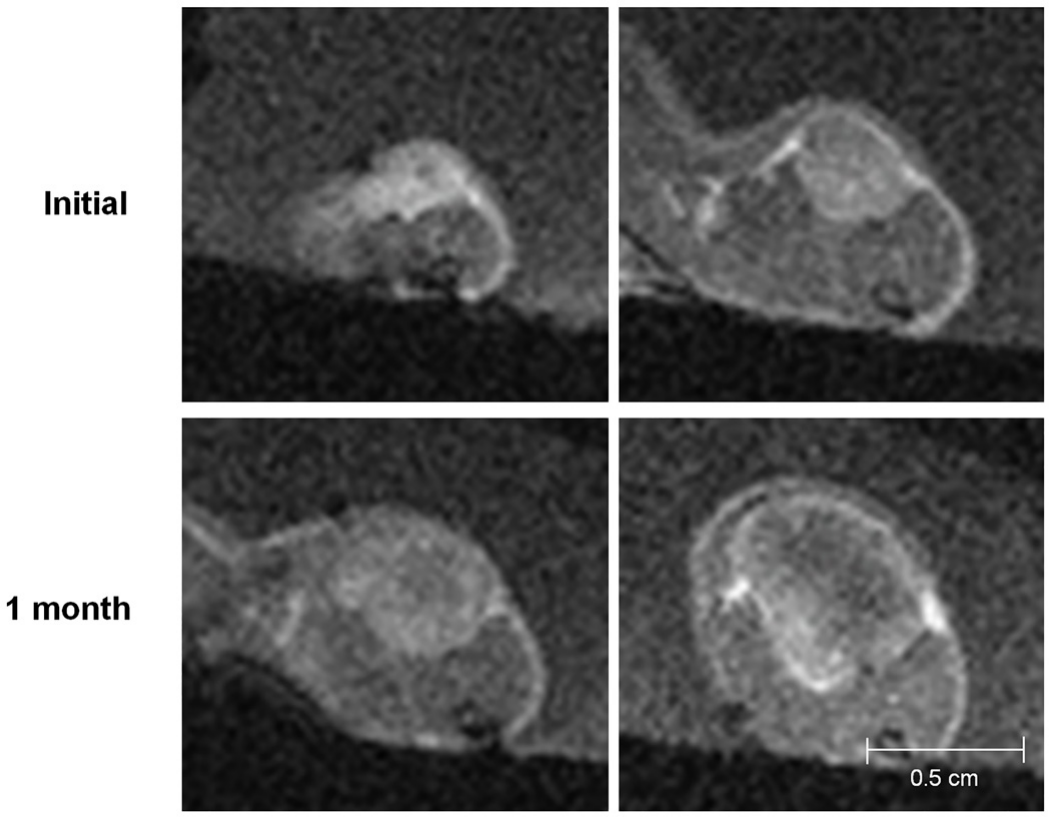
Initial and post-treatment MR images of the antiangiogenic therapy (AAT) group. Tumors in the AAT group mildly increase in size and show a better-perfused state with enhancement than the tumors in the control or radiation therapy group.

**Fig 5:**
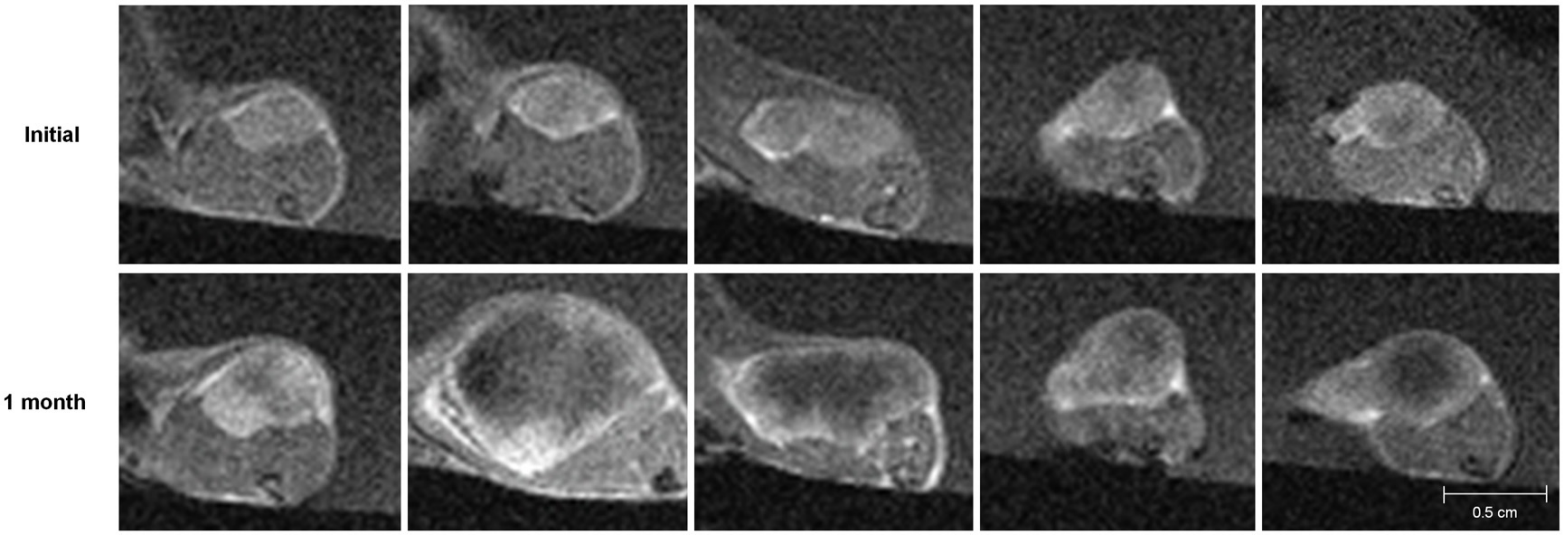
Initial and post-treatment MR images of the radiation therapy group. The tumors increase in size and show extensive central necrosis with low signal intensity and peripheral enhancement.

**Fig 6:**
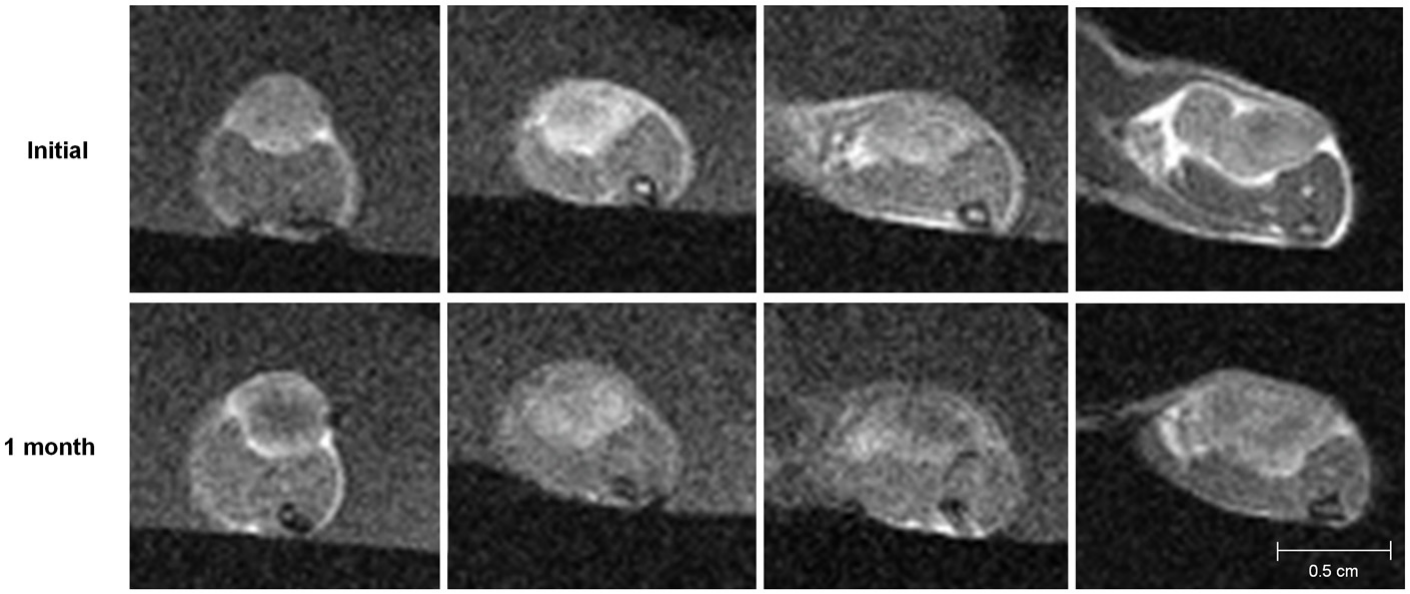
Initial and post-treatment MR images of the combination therapy (AART) group. The tumors in the AART group minimally increase and enhance without remarkable necrosis.

The MR images showed the interval changes of the tumors in each group (Figs 3–6). The post-treatment DCE-MR images of the control group showed significant increases in the tumor sizes and extensive poorly enhanced areas that suggested central necrosis. A wide central poorly enhanced area was also noted in the RT group. The tumors in the AAT group treated only with SU11248 showed a better-perfused state with enhancement than the tumors in the control and RT groups.

**Fig 7:**
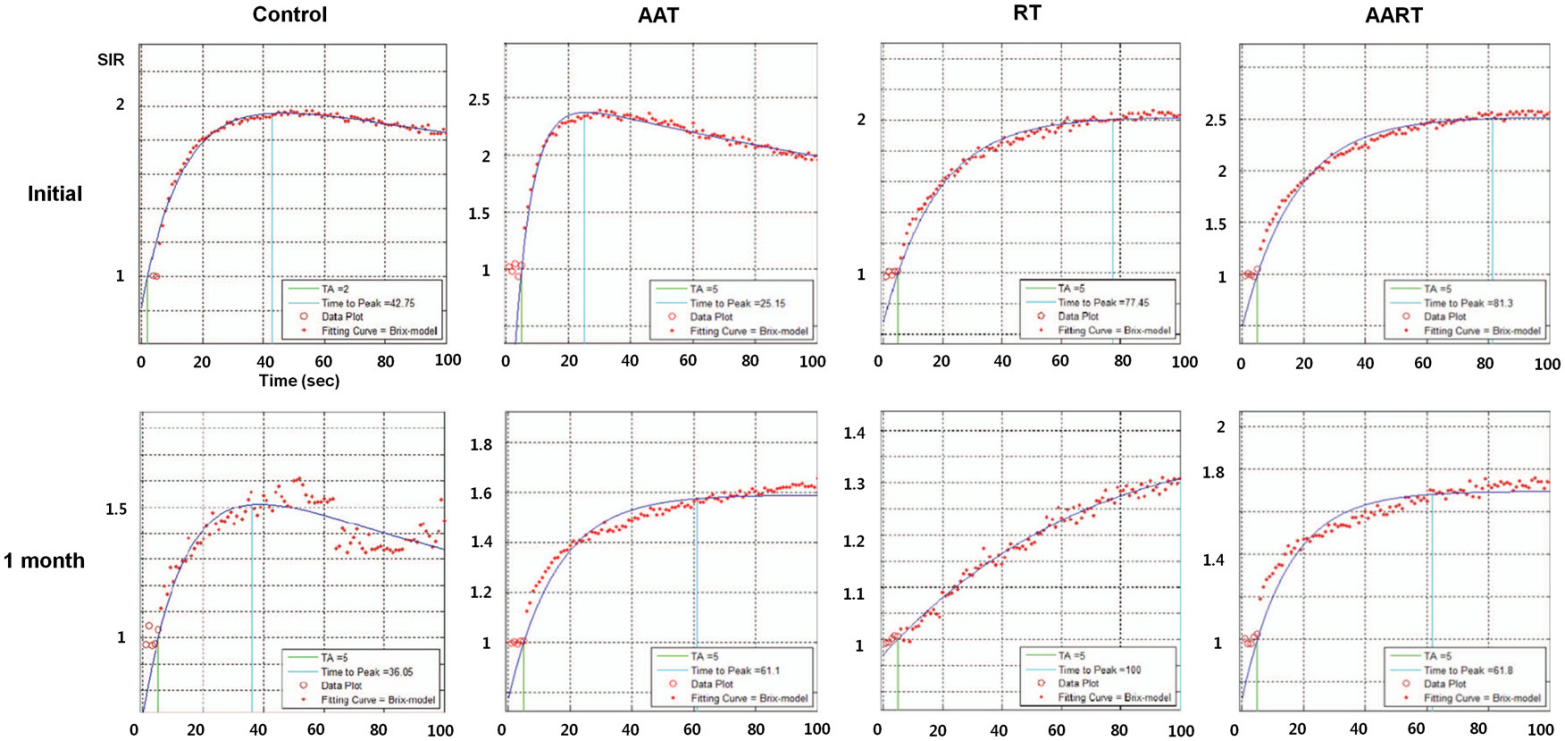
Tumor time-signal intensity curves obtained from the initial and post-treatment MR images of the four cases in each group that represented the interval changes. In the control group, the time-signal intensity curve of the post-treatment MR showed a rapid enhancement and rapid washout pattern. In the AAT group, the enhancement ratios of the tumor are markedly decreased compared to the initial curve even though it shows prolonged enhancement. In the RT group, the signal intensity curve shows a delayed and prolonged enhancement pattern. The rapid wash-in and a plateau pattern of the enhancement in the AART group is similar to that of the tumor managed with AAT alone. However, the enhancement ratio is still higher than those in the AAT case. AAT, antiangiogenic therapy; AART, combination therapy; RT, radiation therapy; SIR, signal intensity ratio—measured by comparison with the initial signal intensity on the precontrast MR images.

To show the time-signal intensity curves at the post-treatment states, the measured serial signal intensities in each tumor were plotted (Fig 7). In the control group, the graph slope, i.e., the speed of enhancement, was steep and gradually decreased after the peak enhancement as Pattern I. On the other hand, in the RT group, the slope of the time-signal intensity curve four weeks after the treatment was flat, and its Pattern III indicated delayed enhancement of the tumor. In the tumors in the AAT group that were treated only with the SU11248, the slope of the time-signal intensity curve after the treatment showed Pattern II, i.e., rapid wash-in and a plateau. Although the tumors in both the RT and AAT groups also showed interval growths with internal necrosis on their DCE-MR images, considering the time-signal intensity curve, the extent of the tumor necrosis seemed lesser in the AAT group than in the RT group. Among the four groups, the tumors in the AART group showed strong and rapid enhancement and a plateau, similar to Pattern II of the AAT group, and had smaller necrotic areas on the MR images than those in the other groups.

### MR perfusion biomarkers

The measured perfusion parameters in the two groups are shown in Table 1. In the AART group, the MER of the tumors was significantly high (p = 0.016), and the S_plat_/TTP, i.e., the time to peak enhancement, which indicates the speed of enhancement, was significantly shorter (p = 0.016) than in the RT group (Table 1). The other parameters, such as K_ep_, K_el_, and A^H^, did not differ significantly between the two groups.

**Table 1:** The five measured perfusion parameters in the RT-alone and AART groups.

Parameter	RT (n = 5)	AART (n = 4)	*P*-value
**K**_**ep**_	0.0002 (0.0001, 0.0049)	0.00006 (0.00003, 0.00012)	0.190
**K**_**el**_	0.0062 (0.0032, 0.0214)	0.0490 (0.0202, 0.0753)	0.063
**S**_**plat**_**/TTP**	0.0049 (0.0035, 0.0061)	0.0107 (0.0078, 0.0142)	0.016
**A**^**H**^	9.40 (1.85, 162.3)	794.05 (201.13, 1958.61)	0.063
**MER**	1.49 (1.354, 1.6126)	1.81 (1.7488, 1.8925)	0.016

The data are medians with the interquartile ranges in parentheses. RT, radiation therapy; AART, SU11248 plus RT; K_ep_ (sec ^-1^), exchange rate constant from the extracellular space back to the plasma in the vascular structure; K_el_ (sec ^-1^), elimination rate constant from the plasma to the renal excretion; S_plat_, signal intensity in the plateau; TTP, time to peak enhancement; A^H^, constant corresponding to the size of the interstitial space; and MER, maximum enhancement ratio.

## Discussion

In this study, we showed that the combination therapy of RT and AAT has a synergistic effect on anticancer treatment using DCE-MR imaging. As a tumor grows, its oxygen demand increases. However, if the cancer vessels cannot supply enough oxygen to the central portion of the tumor, the tumor becomes hypoxic in the central area. Finally, the increased tumor burden and the hypoxic environment that decreases the oxygen-free radicals result in the tumor radio-resistance [15]. AA agents can inhibit PDGF and VEGF signaling and suppress the formation of immature cancer angiogenesis, which is vulnerable to RT. AA agents can also help to retain normal vasculature in the tumor, and thus, reduce the hypoxic area after RT in the tumor [9].

In the RT group, the radiation induced extensive necrosis in the central portion of the tumor and decreased vascular supply. Necrosis with decreased blood supply creates a hypoxic area and decreases the tumoricidal effect of RT by reducing the oxygen-free radicals. However, the tumors in the AAT group showed strong and rapid enhancement with a plateau, although they also showed an interval growth similar to that of the tumors in the RT group. Moreover, the tumors in the AART group were highly enhanced without significant necrosis, and their growth was more significantly suppressed than that of the tumors in the three other groups. These results are consistent with those of previous studies that showed that AA agents suppressed immature angiogenesis and might have helped maintain the normal vasculature in the tumor [7, 9]. By maintaining the tumor perfusion and reducing tumor necrosis, the tumoricidal effects of the oxygen radicals induced by RT could be increased even in the central area of the tumors.

The enhancement curve of the AAT group appeared similar to that of the AART group in terms of the rapid enhancement and maintained plateau even after the treatment. The similar patterns of the time-signal intensity curves on the DCE-MR images imply the similar perfusion patterns of the tumors. Rodgan et al. demonstrated that the microvessel density of a tumor was correlated with the early enhancement of the tumor, and the necrotic tumor portion was correlated with late enhancement [16]. These suggest that the tumors in the AAT group and the AART group might have retained their microvessel density to show rapid and early enhancement; that is, the AA agent might have helped prevent tumor necrosis. Because AA agents can help reduce tumor growth by suppressing the cancer angiogenesis while preserving the normal vasculature, they can eventually help reduce tumor hypoxia [15]. In the other studies, the oxygen levels might have increased in the combination therapy rather than in the RT alone [17–19]. On the other hand, the RT group showed a delayed enhancement pattern with an increased time to peak enhancement. This suggests that the vulnerable cancer vessels in the tumor had been damaged by RT and resulted in more necrotic portions than in the other groups. It has been reported that the time to the peak enhancement of the parotid gland had increased on the DCE-MR images as the vascular permeability decreased and the extracellular extravascular space increased after the RT of the parotid gland [10].

The perfusion changes after each treatment were demonstrated by the DCE-MR images with the expected biomarkers. In this study, the AART group showed a significantly higher S_plat_/TTP (*P*< 0.016) and MER (*P*< 0.016) than the RT group. There are many published studies that attempted to find the quantitative imaging parameters related to the treatment effect on DCE-MR images. The usefulness of K_trans_ and K_ep_ in the assessment of the treatment response has been mentioned in several reports [20–23]. Akisik et al. suggested that the pretreatment K_trans_ for pancreatic tumors might be useful in predicting the treatment response to an AA agent [24]. The effect of anticancer therapy measured by DCE-MR imaging has also been reported with regard to neoadjuvant chemotherapy [25–28] and RT [29–32]. In this study, we showed that one of the explanations of the synergistic effect of AAT on RT could be the retention of the tumor perfusion, which was represented by the higher values of S_plat_/TTP and MER. These results suggest that the early and prolonged enhancement with the plateau seen in the AART group was due to the relatively small portion of the necrosis and from the maintenance of the perfusion in the tumor.

This study had several limitations. First, we used only one type of cancer model, A549, which is a well—differentiated human lung cancer cell line. However, the treatment effect might be diversely demonstrated according to the tumor-specific characteristics such as the expression level of PDGF or VEGF, the angiogenesis potential, the oxygen tension, or the tumor stage. Therefore, the results of this study cannot be directly applied to other types of cancer models. To overcome this limitation, tumor-specific experiments in each cancer cell line and in various clinical settings are needed. Second, as we used only one kind of AA agent, a receptor tyrosine kinase inhibitor, the various mechanisms of AA agents pertaining to RT cannot be sufficiently inferred. Other types of AA agents that target the matrix metalloprotease, inhibit the angiogenesis growth factors and the activation of endothelial cells, and have multiple or unknown mechanisms of action should be investigated for combination with RT [33, 34]. Third, because we have performed MR twice for each mouse before and after the treatment, we could not demonstrate the serial course of tumor changes in each group. To better visualize the perfusion changes in the tumors, follow-up MR during each treatment could be of value. Fourth, although we did not obtain the specimens of the tumors from the mice, the imaging-pathologic correlation could help explain the perfusion changes of the tumors. Finally, this study was not designed to see the tumor changes according to the treatment dose or schedule. Such changes could also affect the tumor condition. A patient’s general health condition could also influence the treatment effect in the clinical setting.

## Conclusion

The combination therapy of an AA agent and RT showed synergistic effects in the anticancer treatment. The AA agent helped maintain the perfusion in the central portion of the cancer mass in the AART group during and after the radiation therapy. The different pattern of the time-signal intensity curve on the DCE-MR imaging in each group implied the perfusion changes of the tumors, which were shown by the perfusion parameters such as S_plat_/TTP and MER on the DCE-MR imaging.

## Supporting Information

S1 Table The measured serial volumes (mm^3^) of the tumors in all mice (n = 13). AAT, antiangiogenic therapy group; AART, combination therapy group; RT, radiation therapy.
(DOCX)
