# Supplementary material for: Synergistic Effect of Anti-Angiogenic and Radiation Therapy: Quantitative Evaluation with Dynamic Contrast Enhanced MR Imaging
Source: PLoS One. 2016 Feb 10;11(2):e0148784. doi: 10.1371/journal.pone.0148784 (PMC4749295; doi:10.1371/journal.pone.0148784)
Supplement: S1 Table — AAT, antiangiogenic therapy group; AART, combination therapy group; RT, radiation therapy. (DOCX) [file pone.0148784.s001.docx]

**Supporting Information**

**S1 Table. The measured serial volumes (mm^3^) of the tumors in all mice (n=13).** AAT, antiangiogenic therapy group; AART, combination therapy group; RT, radiation therapy

| **Group** | **^Day^** | 0 | 2 | 4 | 7 | 9 | 11 | 14 | 16 | 18 | 21 | 23 | 25 |
| --- | --- | --- | --- | --- | --- | --- | --- | --- | --- | --- | --- | --- | --- |
| **Control** | | 88 | 109 | 150 | 170 | 190 | 250 | 280 | 280 | 350 | 350 | 450 | 470 |
|  | | 212 | 226 | 280 | 290 | 310 | 320 | 420 | 460 | 620 | 690 | 700 | 780 |
| **AAT** | | 90 | 100 | 112 | 130 | 133 | 142 | 152 | 160 | 164 | 164 | 168 | 187 |
|  | | 38 | 43 | 48 | 52 | 56 | 55 | 72 | 75 | 80 | 75 | 90 | 102 |
| **RT** | | 99 | 125 | 144 | 140 | 150 | 165 | 170 | 190 | 210 | 230 | 250 | 230 |
|  | | 84 | 96 | 101 | 113 | 120 | 121 | 130 | 188 | 192 | 215 | 222 | 210 |
|  | | 183 | 247 | 256 | 267 | 313 | 350 | 380 | 400 | 420 | 469 | 470 | 628 |
|  | | 126 | 142 | 157 | 186 | 213 | 220 | 250 | 275 | 300 | 420 | 423 | 452 |
|  | | 103 | 108 | 147 | 159 | 167 | 176 | 203 | 200 | 222 | 249 | 285 | 285 |
| **AART** | | 185 | 261 | 270 | 290 | 285 | 320 | 395 | 350 | 317 | 345 | 314 | 285 |
|  | | 125 | 137 | 150 | 155 | 164 | 187 | 190 | 192 | 186 | 174 | 207 | 191 |
|  | | 106 | 131 | 140 | 146 | 164 | 136 | 141 | 160 | 171 | 173 | 184 | 173 |
|  | | 121 | 128 | 128 | 130 | 126 | 121 | 119 | 119 | 113 | 127 | 130 | 135 |
